# The hydrocarbon-bearing clathrasil chibaite and its host–guest structure at low temperature

**DOI:** 10.1107/S2052252518009107

**Published:** 2018-08-08

**Authors:** K. S. Scheidl, H. S. Effenberger, T. Yagi, K. Momma, Ronald Miletich

**Affiliations:** a Institut für Mineralogie und Kristallographie, Universität Wien, Althanstrasse 14, Wien A-1090, Austria; b Geochemical Research Center, Graduate School of Science, The University of Tokyo, 7-3-1 Hongo, Bunkyo-ku, Tokyo 113-0033, Japan; c National Museum of Nature and Science, 4-1-1 Amakubo, Tsukuba, Ibaraki 305-0005, Japan

**Keywords:** chibaite, clathrasils, hydro­carbons, Raman spectroscopy, X-ray diffraction, low temperature

## Abstract

The natural sII-type clathrasil chibaite [chemical formula SiO_2_·(*M* ^12^,*M* ^16^), where *M* *^x^* denotes a guest mol­ecule] was investigated using single-crystal X-ray diffraction and Raman spectroscopy in the temperature range from 273 to 83 K. The O atoms of the structure at room temperature, which globally conforms to space group  [*V* = 7348.9 (17) Å^3^, *a* = 19.4420 (15) Å], have anomalous anisotropic displacement parameters indicating a static or dynamic disorder. With decreasing temperature, the crystal structure shows a continuous symmetry-lowering transformation accompanied by twinning. The intensities of weak superstructure reflections increase as temperature decreases. A monoclinic twinned superstructure was derived at 100 K [*A*2/*n*, *V* = 7251.0 (17) Å^3^, *a*′ = 23.7054 (2), *b*′ = 13.6861 (11), *c*′ = 23.7051 (2) Å, β′ = 109.47°]. The transformation matrix from the cubic to the monoclinic system is *a_i_*′ = (½ 1 ½ / ½ 0 −½ / ½ −1 ½). The *A*2/*n* host framework has Si—O bond lengths and Si—O—Si angles that are much closer to known values for stable silicate-framework structures compared with the averaged  model. As suggested from band splitting observed in the Raman spectra, the [5^12^]-type cages (one crystallographically unique in , four different in *A*2/*n*) entrap the hydro­carbon species (CH_4_, C_2_H_6_, C_3_H_8_, *i*-C_4_H_10_). The [5^12^6^4^]-type cage was found to be unique in both structure types. It contains the larger hydro­carbon mol­ecules C_2_H_6_, C_3_H_8_ and *i*-C_4_H_10_.

## Abstract 2

The crystal structure of chibaite (a natural clathrasil with an MTN-type framework structure) and its structural evolution with decreasing temperature from 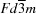 space-group symmetry to *A*2/*n* were investigated with single-crystal X-ray diffraction and Raman spectroscopy.

## 1. Introduction   

Clathrates are inclusion compounds which are built up from topologically sub-nanoporous host frameworks that entrap guest atoms and mol­ecules of suitable size into cage-like voids. Various clathrates have gained interest because of their application as potential materials for gas storage and gas separation of H_2_ as well as the greenhouse gases CO_2_ and CH_4_ (Burggraaf *et al.*, 1998 ▸; Algieri *et al.*, 2003 ▸; Min *et al.*, 2003 ▸; Navrotsky *et al.*, 2003 ▸; van den Berg *et al.*, 2004 ▸, 2005 ▸; Hong *et al.*, 2005 ▸; House *et al.*, 2006 ▸; Di Profio *et al.*, 2007 ▸; Dong *et al.*, 2008 ▸; Kanezashi *et al.*, 2008 ▸; Zheng *et al.*, 2008 ▸; Eslamimanesh *et al.*, 2012 ▸). The cages have small pore openings and so guest species are trapped inside the crystal structure; thus, diffusion of the atoms and mol­ecules out of the cages is slow in comparison with most microporous zeolite structures (Binder *et al.*, 2013 ▸; Hu *et al.*, 2014 ▸; Fujiyama *et al.*, 2015 ▸; O’Malley *et al.*, 2016 ▸). Clathrate frameworks consisting of only SiO_2_ are referred to as clathrasils. They exhibit structural analogs to H_2_O-ice phases which are also observed for several other SiO_2_ phases (Kamb, 1965 ▸). The three clathrasils found in nature to date, melanophlogite (MEP-framework topology) (Gies, 1983 ▸; Nakagawa *et al.*, 2001 ▸), chibaite (MTN-framework topology known from the zeolites ZSM-39, CF-3, dodecasil-3C and holdstite) (Baerlocher *et al.*, 2007 ▸; Momma *et al.*, 2011 ▸) and bosoite (DOH-framework) (Momma *et al.*, 2011 ▸, 2014 ▸), were proven to be isostructural with the gas–hydrate structure types sI, sII and sH. Clathrasils are found in marine sediments related to low-temperature hydro­thermal processes of convergent plate boundaries (Momma *et al.*, 2011 ▸; Momma, 2014 ▸; Likhacheva *et al.*, 2016 ▸).

Following the structural description reported (Brooks *et al.*, 1984 ▸; Lu *et al.*, 2007 ▸; Momma *et al.*, 2011 ▸), the sII-type representative chibaite studied here is built up from a three-dimensional framework of corner-sharing [SiO_4_] tetrahedra forming two different cage types, the smaller pentagon-dodecahedra [5^12^]-cages and the larger hexadecahedra [5^12^6^4^]-cages (superscripts indicate the number of pentagonal and hexagonal faces of the cage). The cages are reported to incorporate the guest mol­ecules N_2_, CO_2_ and small hydro­carbons including methane (CH_4_), ethane (C_2_H_6_), propane (C_3_H_8_) and isobutane (*i*-C_4_H_10_) (Momma *et al.*, 2011 ▸; Likhacheva *et al.*, 2016 ▸). Only hydrogen bonds and weak van der Waals inter­actions act between the guest mol­ecules and the framework. These mol­ecules presumably serve as templates during the crystallization of the clathrasil host structures in order to stabilize the sub-nanoporous framework that is known for gas hydrates (Gies *et al.*, 1982 ▸; Navrotsky *et al.*, 2003 ▸). Analogous to gas hydrates, the occurrence of the smaller hydro­carbons CH_4_ and C_2_H_6_ promotes the crystallization of sI structures, and the addition of the larger hydro­carbons C_3_H_8_ and *i*-C_4_H_10_ promotes the crystallization of sII and sH structures (Davidson *et al.*, 1986 ▸; Kvenvolden, 1995 ▸; Lu *et al.*, 2007 ▸).

The ideal formula of the apparent cubic chibaite is SiO_2_·(*M* ^12^,*M* ^16^), with *M^x^* being the guest mol­ecule in the corresponding *x*-faced polyhedral cage. The highest possible space-group symmetry of the sII framework (MTN-framework topology) is 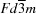 (Könnecke *et al.*, 1992 ▸). However, the true symmetry appears to depend on temperature (Gies, 1983 ▸; Könnecke & Fuess, 1995 ▸), pressure (Tribaudino *et al.*, 2010 ▸) and the type of guest species as well as its orientation (Momma *et al.*, 2013 ▸; Momma, 2014 ▸). Various low-symmetry structures of dodecasil-3C, the synthetic analog of chibaite, correspond in an inconsistent fashion to subgroups of the ideal 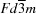 framework (Gies, 1984 ▸; Chae *et al.* 1991 ▸; Könnecke *et al.*, 1992 ▸; Könnecke & Fuess, 1995 ▸; Knorr & Depmeier, 1997 ▸). Moreover, several studies describe a temperature-induced phase transition of dodecasil-3C (Gies, 1984 ▸; Ripmeester *et al.*, 1988 ▸; Tse *et al.*, 1993 ▸; Könnecke & Fuess, 1995 ▸). The displacive distortions are presumably induced by the distribution and ordering of the entrapped guest mol­ecules.

The aim of this study was to investigate the crystallography of chibaite single crystals from a new locality in Nagano Prefecture, Japan. A detailed study focused on formation and chemical characterization is in progress. By applying low-temperature conditions in the range from 293 to 83 K, the structural evolution of this complex host–guest clathrasil structure is described.

## 2. Materials and methods   

The studies were performed on two small (111)-oriented double-sided polished crystal platelets (each of about 80 × 50 × 40 µm in size) prepared from a natural chibaite crystal. The specimens were carefully inspected with regard to crystal quality, optical homogeneity and crystal impurities using the highest magnification (120×) of a stereomicroscope at room temperature (RT). No domains or related microstructures nor inclusions nor birefringence were observed under polarized light.

Raman spectroscopy was performed using a Jobin–Yvon Horiba LabRam HR800 instrument equipped with a CCD detector, operated in confocal mode. The measurements were carried out using a 50× long-working-distance objective and setting a grating with 1800 grooves mm^−1^. The spectral resolution was calibrated with the Rayleigh line of the laser resulting in a resolution better than 0.5 cm^−1^. The sample was excited with a 532 nm laser, providing about 34 mW on the sample surface. Spectra were collected at RT as well as at low temperatures (LT) down to 83 K. LT measurements were performed using a Linkam FTIR 600 liquid nitro­gen cooling stage, which enabled temperature (T) control with an accuracy better than ±2 K. Raman spectra were collected in the frequency range from 60 to 3600 cm^−1^ with 60 s counting time and two accumulations. In order to evaluate band positions and full widths at half maxima (FWHM), the recorded bands were fitted with the program *PeakFit* (Systat Software, 2007 ▸) after subtracting the background by assuming Lorentzian–Gaussian band shapes and applying the Gauss–Lorentz area method.

Afterwards, a platelet from the same crystal used for the Raman measurements was selected and mounted on a glass fiber for single-crystal X-ray diffraction (sXRD) investigations. Precise unit-cell parameters at RT were determined from the peak profiles of strong sXRD Bragg reflections. These were measured using the eight-position centering technique with a Huber 5042 four-circle diffractometer (non-monochromated Mo radiation, conventional sealed tube source). The setting angles of 22 non-equivalent reflections in the 2θ range from 7 to 30° were determined by applying the peak-fitting algorithm implemented in the *SINGLE* software (Angel & Finger, 2011 ▸).

Both sXRD intensity data sets and unit-cell parameters under LT conditions were measured using a StoeStadiVari diffractometer, equipped with a Dectris Pilatus 300K pixel detector and operated with monochromated Mo *K*α radiation from a 100 W air-cooled Incoatec IμS micro-focus X-ray tube (50 kV, 1 mA). The temperatures 293, 273, 250, 200, 150 and 100 K were maintained using the flowing N_2_ gas cooling device from Oxford Cryosystems Ltd, which is stable within ±0.1 K. ω scans at different χ and φ positions with a scan width of 0.5° were used to optimize the coverage of the full sphere of the reciprocal space. A detector-to-crystal distance of 60 mm was set for all measurements. Data processing (indexing, integration, Lorentz polarization correction) was performed using the *X-AREA* software (Stoe & Cie, 2002 ▸). Owing to the low absorption coefficient of the sample material (μ = 6.24 cm^−1^), only a multi-scan absorption correction by means of frame scaling was applied. Details of the instrumental settings for individual intensity data collections and information on the data processing are summarized in Table 1 ▸. Structure refinements were performed using the program *SHELXL97* (Sheldrick, 2008 ▸, 2015 ▸) and the *OLEX* ^2^ software (Dolomanov *et al.*, 2009 ▸) after data reduction with the *X-AREA* software (Stoe & Cie, 2002 ▸). Neutral atomic scattering factors were taken from the *International Tables for X-ray Crystallography* (Maslen *et al.*, 2004 ▸). The linear thermal expansion coefficient fitted to the series of unit-cell-volume data points followed the Kroll formalisms (Kroll *et al.*, 2012 ▸) implemented in the software *EoSFit7* (Angel *et al.*, 2014 ▸).

## 3. Results   

### 3.1. Guest mol­ecules and their distribution   

**Figure 1:**
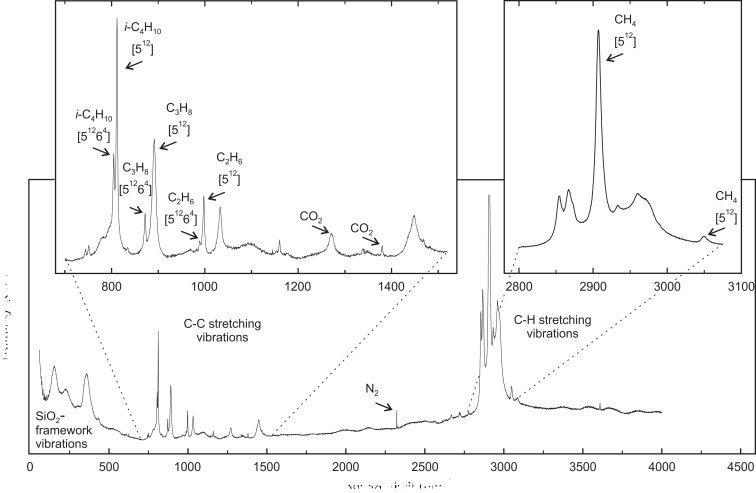
Raman spectrum of chibaite under ambient conditions. The two inserts represent the enlarged cut-outs of 700–1500 cm^−1^ and 2800–3100 cm^−1^ containing the hydrocarbon C—C and C—H stretching vibrations. The labeling of the allocated major bands corresponds to the respective mol­ecule and the cage types (see Table 2 ▸) (Sum *et al.*, 1997 ▸; Charlou *et al.*, 2004 ▸; Tribaudino *et al.*, 2008 ▸; Klapp *et al.*, 2010 ▸; Momma *et al.*, 2011 ▸; Likhacheva *et al.*, 2016 ▸).

**Figure 2:**
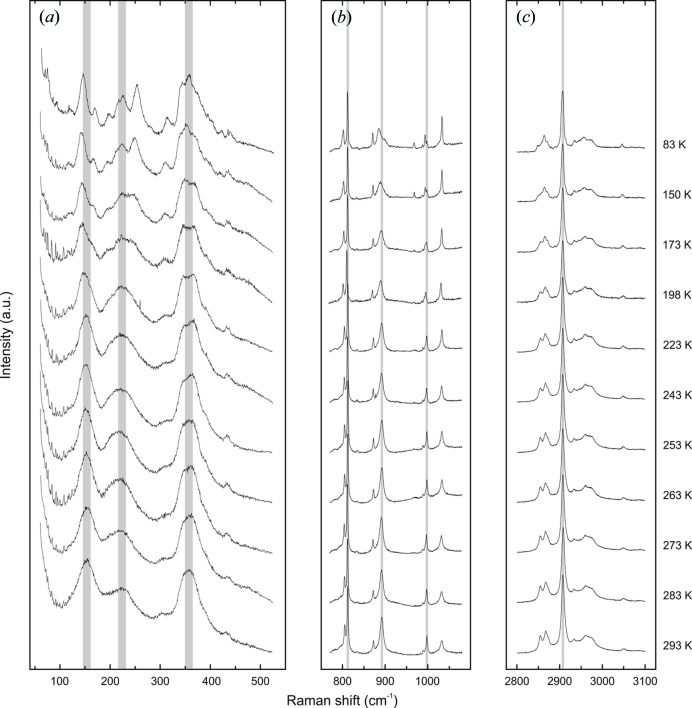
Selected ranges of the Raman spectra between 293 and 83 K. (*a*) SiO_2_-framework vibrations (60–500 cm^−1^), (*b*) C—C stretching vibrations (770–1080 cm^−1^), (*c*) C—H stretching vibrations (2800–3100 cm^−1^).

**Figure 3:**
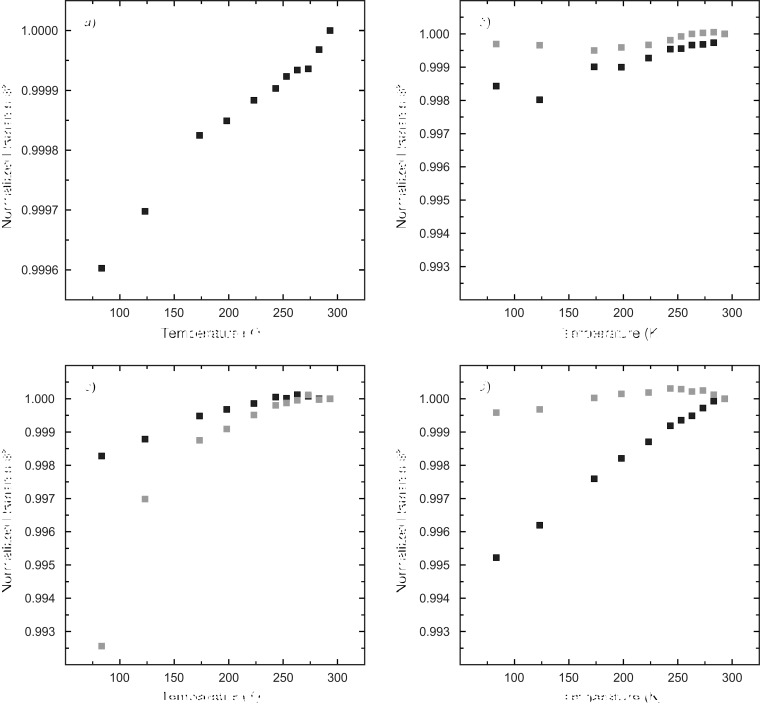
Raman shift of the bands assigned to the individual guest mol­ecules and their evolution with decreasing T (relative to RT): (*a*) CH_4_, (*b*) C_2_H_6_, (*c*) C_3_H_8_ and (*d*) *i*-C_4_H_10_. Black squares correspond to the bands assigned to the [5^12^] cage, gray squares to those of the [5^12^6^4^] cage. The ESDs are smaller than the symbol size.

Raman spectroscopy has proven to be a convenient method for the characterization of guest mol­ecules in clathrate phases. It not only allows for the identification of the mol­ecular guest species (Yagi *et al.*, 2007 ▸; Tribaudino *et al.*, 2008 ▸; Bourry *et al.*, 2009 ▸), but also their assignment to distinct cage types (Sum *et al.*, 1997 ▸; Subramanian & Sloan, 2002 ▸; Hirai *et al.*, 2010 ▸; Momma *et al.*, 2011 ▸). Moreover, minor changes in the local environment that can be attributed to distinct temperature and pressure conditions are recognizable (Shimizu, 2003 ▸; Machida *et al.*, 2006 ▸; Gatta *et al.*, 2014 ▸; Likhacheva *et al.*, 2016 ▸). Fig. 1 ▸ shows the Raman spectrum of chibaite at RT. The positions of the Raman bands belonging to the guest mol­ecules are listed in Table 2 ▸. Fig. 2 ▸ shows the series of Raman spectra at LT. Fig. 3 ▸ displays the variation of the band positions assigned to the hydro­carbons as a function of T.

The recorded Raman spectra contain three spectral ranges of particular interest: (i) the SiO_2_-framework vibrations between 60 and 700 cm^−1^, (ii) the C—C stretching vibrations between 700 and 1100 cm^−1^, and (iii) the C—H stretching vibrations between 2800 and 3100 cm^−1^ (Fig. 1 ▸). The analysis of the spectral range assigned to the framework vibrations has not yet been discussed in any previous studies. The spectral ranges of the C—C and C—H stretching vibrations were not only used to identify hydro­carbon guest mol­ecules in gas hydrates and clathrasils, but also to allocate them to distinct cages within the host framework. In accordance with Kolesov & Geiger (2004 ▸), the positions of the Raman bands assigned to the distinct guest mol­ecules are shifted to lower Raman shifts relative to the bands assigned to the same mol­ecules in the gas phase because of the inter­action between the mol­ecules and the framework. The different sizes of the two cages influence the vibration of the mol­ecules to a different degree. The inter­actions between the framework and mol­ecules are stronger within the small [5^12^] cages, causing band positions with higher Raman shifts with respect to those in the larger [5^12^6^4^] cages. Consequently, if the mol­ecules are distributed in both cage types, the Raman bands are split (Subramanian & Sloan, 2002 ▸).

The Raman bands of this study were assigned to methane (CH_4_) (Sum *et al.*, 1997 ▸), ethane (C_2_H_6_) (Klapp *et al.*, 2010 ▸), propane (C_3_H_8_) (Sum *et al.*, 1997 ▸), iso­butane (*i*-C_4_H_10_) (Klapp *et al.*, 2010 ▸), CO_2_ (Charlou *et al.*, 2004 ▸) and N_2_ (Tribaudino *et al.*, 2008 ▸) (Table 2 ▸, Fig. 1 ▸). The C—H stretching vibration *ν*_1_ of CH_4_ shows only one single band at 2907.5 (5) cm^−1^ as a result of the mol­ecule being exclusively entrapped in the [5^12^] cages (Momma *et al.*, 2011 ▸; Likhacheva *et al.*, 2016 ▸). The band at 3049.8 (5) cm^−1^ is assigned to the overtone of the C—H asymmetric bending vibration belonging to the CH_4_ mol­ecule (2*ν*_2_) (Momma *et al.*, 2011 ▸). Unfortunately, it was not possible to assign the residual C—H stretching vibrations to the larger hydro­carbons because of their complex spectra and extensive overlapping. The bands assigned to the C—H vibration of CH_4_ are not split, whereas the bands belonging to the C—C stretching vibrations of C_2_H_6_, C_3_H_8_ and *i*-C_4_H_10_ are split into two components, indicating that the three larger hydro­carbons are located in both cage types.

The relative distribution of the guest mol­ecules was estimated according to the ratios of the integrated intensities of the Raman bands. The intensities of the bands assigned to the mol­ecules located in the smaller [5^12^] cages are always higher than those belonging to the mol­ecules located in the larger [5^12^6^4^] cages. The intensity ratios of the [5^12^] cage to the [5^12^6^4^] cage are: ∼6.6:1 for C_2_H_6_, ∼6:1 for C_3_H_8_ and ∼2:1 for *i*-C_4_H_10_. In the sII-type framework, the number of [5^12^] cages relative to [5^12^6^4^] cages is 2:1. Thus, the intensity ratio suggests that C_2_H_6_ and C_3_H_8_ occupy the small cages about three times more often than the large cages; in contrast, the larger hydro­carbon *i*-C_4_H_10_ seems to be distributed equally between the two different cage types.

The Raman bands at 1271.9 (5) and 1380.0 (5) cm^−1^ are assigned to the Fermi dyads (*ν*_c−_) and (*ν*_c+_) of the CO_2_ vibrations, and the band at 2322.3 (5) cm^−1^ to N_2_ (Tribaudino *et al.*, 2008 ▸). No splitting was observed for either species.

### 3.2. Evolution of the Raman spectra under LT conditions   

Figs. 2 ▸ and 3 ▸ show the changes of the Raman spectra with T from 293 to 83 K. At RT, the SiO_2_-framework vibrations yield three broad but prominent bands located at ∼155, ∼230 and ∼360 cm^−1^, and two small bands at ∼310 and ∼430 cm^−1^. As T decreases, these bands sharpen and evolve into several split components and shoulders of smaller FWHMs, indicating a lowering of the symmetry. The spectral evolution with decreasing T does not reveal any obvious discontinuity indicative of a distinct and spontaneously occurring phase transition. Results indicate a continuous distortion of the host framework which becomes stronger as T decreases. The occurrence of birefringence and the formation of crystallographic domains on a microscopic scale at *T* ≤ 123 K are a further proof for the lowering of symmetry at LT.

The Raman shifts and intensities of the Raman bands assigned to the guest mol­ecules decrease with increasing T (Figs. 2 ▸ and 3 ▸) as a result of the damped resonance amplitude and reduced vibration energy of the mol­ecules. The redshifts of the bands assigned to the C—C vibrations vary with the presence of different hydro­carbons (C_2_H_6_, C_3_H_8_ and *i*-C_4_H_10_) and cage types (Fig. 3 ▸). The CH_4_ mol­ecules, which are located exclusively in the [5^12^] cages, reveal a small but near-linear shift. This is caused by a minor change in the inter­action between the framework and the mol­ecule in the center of the cage. Therefore, the relative slope of the Raman shift of CH_4_ from 293 to 83 K is almost negligible compared with the spectral changes of the other hydro­carbon mol­ecules. Owing to the smaller kinetic diameter of C_2_H_6_ compared with *i*-C_4_H_10_ and C_3_H_8_, the influence of the inter­action with the surrounding SiO_2_-framework is much less significant and leads to a gentler slope. The change of the Raman shifts of C_2_H_6_ and *i*-C_4_H_10_ with T is more prominent for the mol­ecules located in the [5^12^6^4^] cage compared with those located in the [5^12^] cage, which differs from what can be observed for C_3_H_8_.

### 3.3. Lattice metrics and space-group symmetry   

The optical properties of the crystals under crossed polarizers suggest a cubic symmetry, which is supported by the measurements performed using the high-resolution Huber diffractometer resulting in cubic lattice metrics. The parameters and their estimated standard deviations (ESDs) obtained from the symmetry-unconstrained triclinic refinement at RT are: *a* = 19.442 (2), *b* = 19.445 (2), *c* = 19.443 (2) Å, α = 90.013 (9), β = 90.010 (11), γ = 90.006 (11)° and *V* = 7350.4(1.4) Å^3^. The values of all unit-cell axes are equivalent within their ESDs and the deviation of the angles from the ideal 90° is less than 1.5σ, *i.e.* <0.013°. The refinement constrained to cubic symmetry finally yielded: *a* = 19.4447 (10) Å and *V* = 7351.9(1.2) Å^3^. The LT data collected on the StadiVari system were also carefully evaluated with respect to a potentially lower symmetry. However, no significant deviation from the cubic cell metric is observed at any T measured down to 100 K (Table 1 ▸). The unit-cell volume steadily decreases without any detectable discontinuity. There is no evidence for a discrete phase transition or any sudden structural transformation associated with a change in the cell volume. The corresponding linear expansion coefficient determined by linear regression on the unit-cell volumes yields α = 7.8 (2) × 10^−6^ K^−1^.

First, all diffraction patterns were indexed based on the cubic unit-cell axes according to these findings. The systematic absences of the measurements at RT are consistent with the extinction group 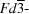, which is in accordance with earlier structure refinements of dodecasil-3C in the space groups 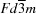 and 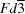 (Gies, 1984 ▸; Könnecke *et al.*, 1992 ▸). Although the reliability factors of the RT structure refinement of an analogous holohedral cubic model 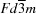 with 46 variable parameters provided quite reasonable results (*R* = 0.055, *wR* = 0.133, GooF = 1.11), the respective factors progressively increase with decreasing T (Table 1 ▸). When comparing the corresponding reflection conditions of the individual data sets, it appears that the *d*-glide plane is consistently violated at LT. The intensities of systematically forbidden reflections become stronger and their numbers increase continuously from 0 at RT to 585 reflections at 100 K (Table 1 ▸). The reflection statistics of the data reduction, as well as a careful inspection of the reciprocal space, do not indicate any violation of the *F* centering. However, the averaging of symmetry-equivalent reflections based on cubic Laue symmetry yields unusually high values of *R*_int_, again increasing steadily from *R*_int_ = 0.096 at RT to 0.33 at 100 K.

**Figure 4:**
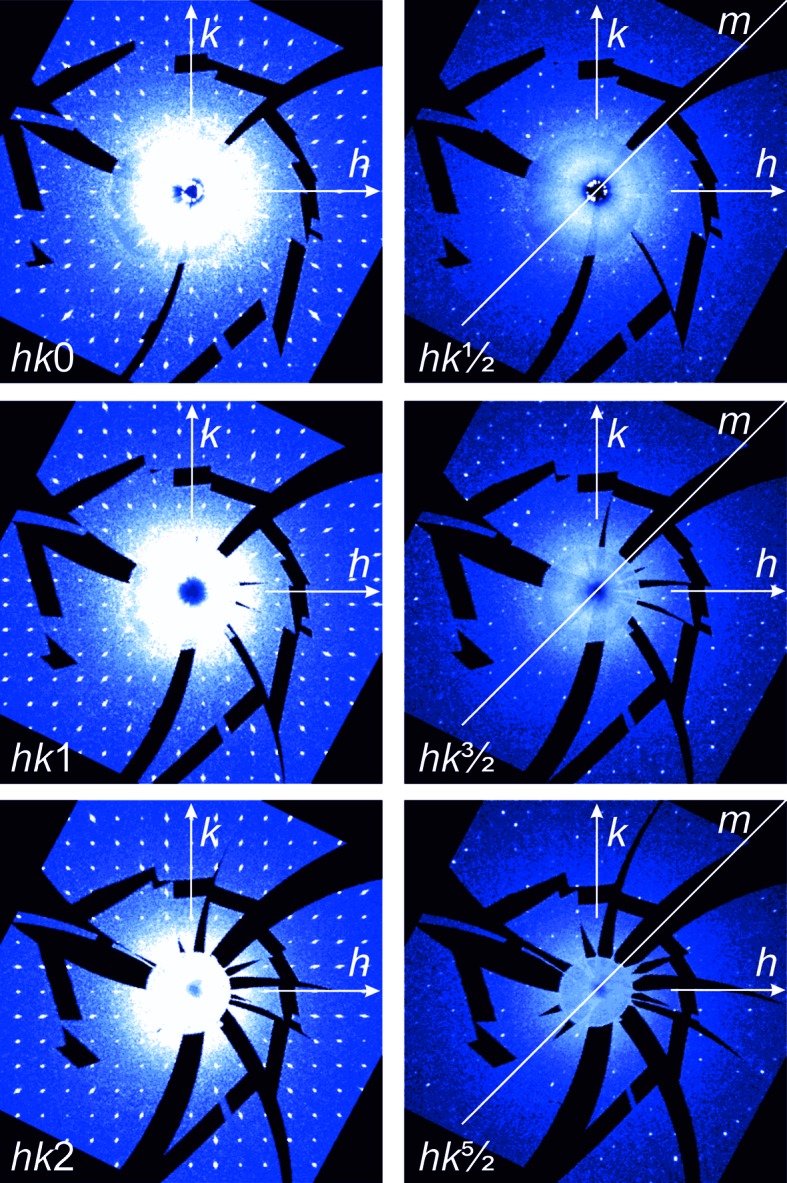
Reciprocal space of the sXRD data measured at 100 K reconstructed in sections perpendicular to c* (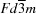 setting). The image cutouts correspond to −14 ≤ *h*, *k* ≤ 14, each centered at 00*l*. The layers *hk*0, *hk*1 and *hk*2 represent only reflections occurring in the cubic parental structure. The weak superstructure reflections (in layers with *l* = 1/2, 3/2, 5/2,… only) indicate Laue symmetry 2/*m* and twinning (marked by the twin plane *m*).

**Figure 5:**
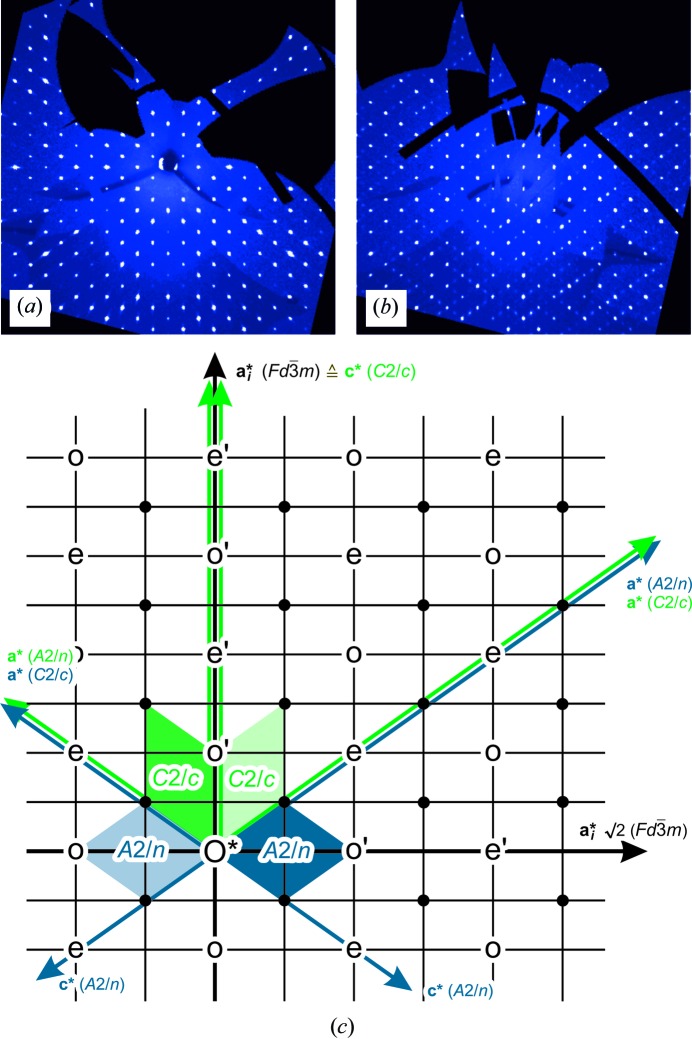
Reconstructed reciprocal space at 100 K; the orientation parallel to the (*hhl*) (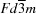 setting) and (010) planes (*A*2/*n* and *C*2/*c* setting), respectively. (*a*) Section centered in 000 (= O*). (*b*) Section centered in 220. (*c*) Axes directions are indicated in a corresponding schematic drawing of the reciprocal lattice. The cubic basis vectors (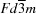) are represented by black arrows, and the reduced monoclinic *A*2/*n* and the standard monoclinic *C*2/*c* settings are given in blue and green, respectively. Twin domains following the twin law for the *A*2/*n* setting (0 0−1 / 0 1 0 / −1 0 0) are shaded. Reciprocal-lattice points corresponding to weak superstructure reflections are marked by small black dots. o and e mark strong reflections occurring in odd planes only or even planes only, respectively; e′ and o′ mark reflections missing in the equatorial plane for space group 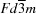 according to the reflection conditions of the *d*-glide plane (*k* + *l* = 4*n* for 0*kl*).

Reconstructed diffraction patterns of the reciprocal space reveal weak superstructure reflections, which show increasing intensity with decreasing T (Fig. 4 ▸). The superstructure reflections occur in all three main directions along a*, b* and c* with respect to the cubic 19.4 Å basis vectors, *i.e.* in the corresponding 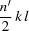, 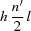 and 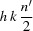 sections with *n′* = 2*n* + 1. Sections parallel to *hk*0 in a sequence along c* reveal the monoclinic Laue symmetry 2/*m*. This seems to be most obvious regarding the subset of weak intensities in the layers where *l* = 2*n* + 1 (Fig. 4 ▸). The orientation of the unique monoclinic mirror plane matches the reciprocal *hhl* lattice-plane direction. At the same time, integral as well as zonal extinctions within the reciprocal-space planes parallel to *hhl* suggest a base-centered monoclinic supercell and the existence of a glide plane (Fig. 4 ▸). Together with the twinning according to
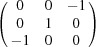
of the monoclinic setting, the arrangement of the reflection conditions suggests the space-group symmetry *A*2/*n* (*i.e.* non-standard setting of *C*2/*c*, which requires β = 125.26°). Consequently, the transformation matrix from the cubic 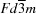 to the monoclinic *A*12/*n*1 setting is
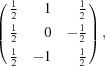
with *a*′ = *c*′ = *a*_cub_/2 × 2^1/2^ × 3^1/2^ = 23.7051 Å, *b*′ = *a*_cub_/2 × 2^1/2^ = 13.6861 Å and β′ = 2arctan 2^1/2^ = 109.47° (Figs. 5 ▸ and 6 ▸). The unit-cell volume of the monoclinic *A*-centered cell is equivalent to that of the cubic *F*-centered cell.

### 3.4. The *A*2/*n* structure model at 100 K   

The data set collected up to sinθ/λ = 0.771 Å^−1^ at 100 K [*i.e.* 93896 individual reflections, merged to a set of 7878 unique data with *F*_o_ > 4σ(*F*_o_) classified as observed] yields an acceptable value of *R*_int_ = 0.138 for the Laue symmetry 2/*m*. After several refinement cycles of the 52 framework-atom positions, a total of 16 extra-framework positions were extracted from the difference Fourier summation. The anisotropic displacement parameters (ADPs) *U_ij_* for all framework atoms were refined. The refinement of, in total, 533 parameters, including the twin fraction with the above-mentioned twin law, converged to an *R*_1_ value of 0.120 with a final residual electron density of ±2.2 e Å^−3^ (Table 1 ▸). The values of the refined atomic parameters of the twinned *A*2/*n* model are listed in Table S1 of the supporting information. A summary of the ranges of the bond distances and the bond angles between framework atoms is provided in Table 3 ▸.

**Figure 7:**
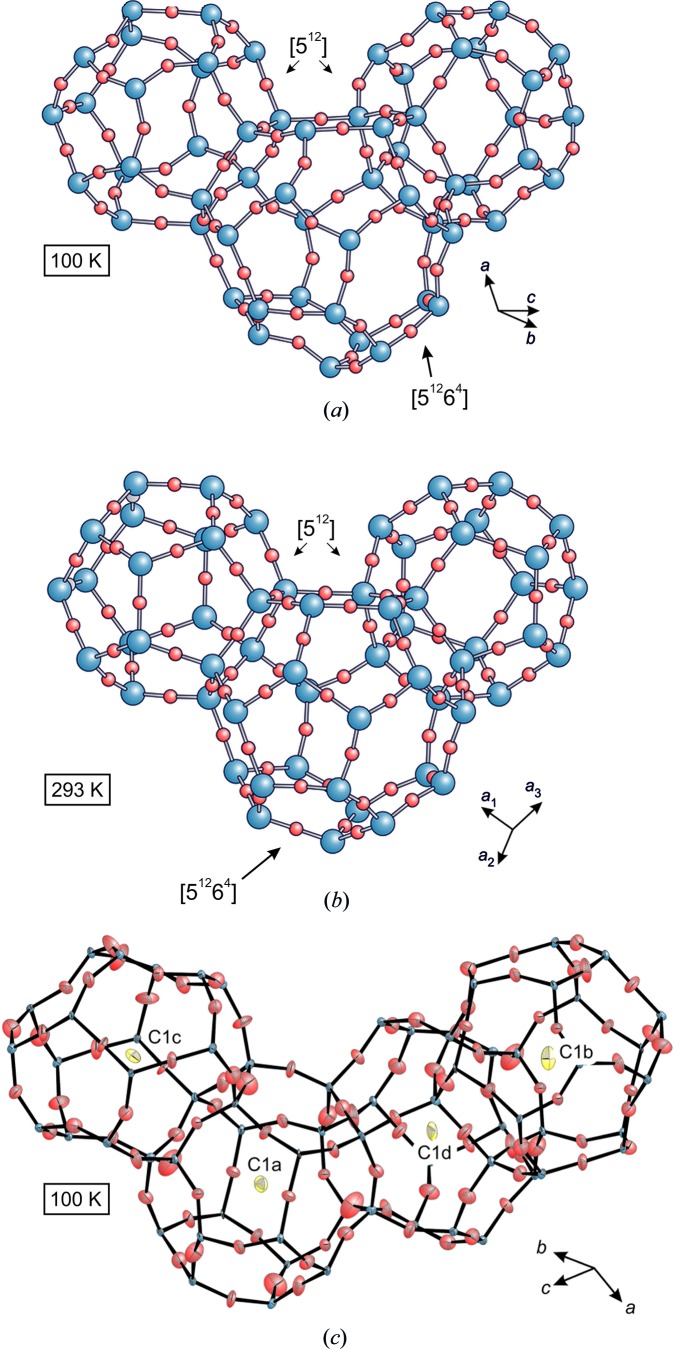
Comparison of the [5^12^]- and [5^12^6^4^]-type cages at (*a*) *T* = 100 K (*A*2/*n*), and (*b*) 293 K (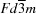). Small red spheres represent the O atoms, and the large blue spheres are the Si atoms. Several Si—O—Si angles bend significantly in the LT form (100 K) in comparison with that at RT (293 K). (*c*) The four crystallographically different [5^12^]-type cages (100 K, *A*2/*n*), inter­connected by sharing common five-membered ring units; the symmetry-released C1 atoms (labeled C1a, C1b, C1c, C1d) are located in the cage centers. All atoms are shown as 50% probability ellipsoids recalculated from the corresponding *U_ij_* values. As a result of the strong bending of the Si—O—Si bond angles, the individual cages are subject to significant distortions at LT and the deviation from the (average) 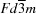 symmetry is evident.

**Figure 8:**
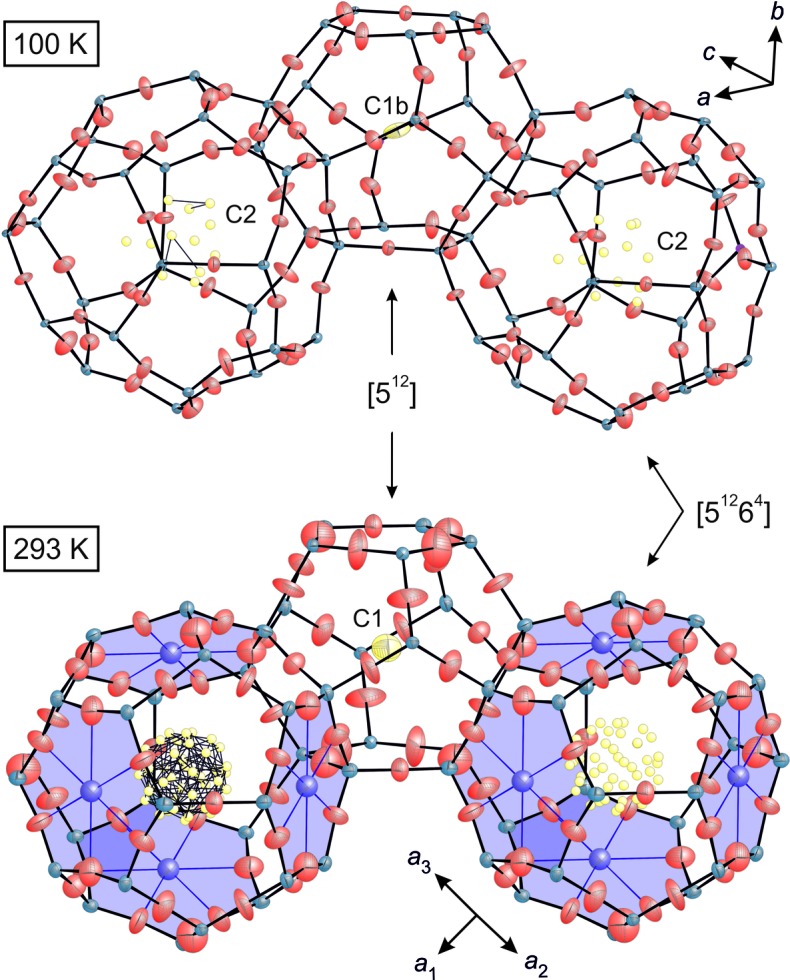
Detail of the chibaite framework at *T* = 100 K (above) and 293 K (below): the [5^12^]- and [5^12^6^4^]-type cages are linked *via* five-membered rings. All framework atoms (Si: blue, O: red) and the fully occupied C1 position (yellow) are shown as 50% probability ellipsoids determined from the corresponding *U_ij_* values. All partially occupied C2 positions (small yellow spheres) are located in the central region of the [5^12^6^4^] cage. The purple areas mark the six-membered ring units with the Na atoms and Na—O bonds (in purple).

During symmetry lowering to *A*2/*n*, the crystal structure develops four crystallographically independent pentagon-dodecahedral [5^12^] cages (Fig. 7 ▸); however, for the larger hexadecahedra [5^12^6^4^] cages, a single type is maintained. The C1 positions are assigned to the four [5^12^] cages (labeled C1a to C1d), and a total of 12 C2 positions are located inside the [5^12^6^4^] cage (labeled C2a to C2l) (Fig. 8 ▸). The four C1 sites correspond to the four individual [5^12^] cage centers, each located either on an inversion center or on a twofold axis. First, their site occupancy factors (SOFs) were released in the refinement, while the *U*_iso_ values were fixed to 0.05 Å^2^. As the SOFs converged to 1.0 within their ESDs, the refinement procedure was changed: the SOFs of the four C1 atoms were kept fixed and their ADPs were allowed to vary. The position of the C1 atoms likely represents the barycenter of the mol­ecules CH_4_, C_3_H_8_, *i*-C_4_H_10_ and CO_2_. The inter­atomic distances of these C1 centers to the framework O atoms is ≥3.57 Å. As a result of the low and rather diffuse residual electron density in the immediate vicinity of the C1 centers, it is not possible to localize further distinct positions inside the [5^12^] cages. The mol­ecules might be statistically distributed without any coherent preferred alignment across the crystal. The SOFs of the 12 assigned C2 positions inside the [5^12^6^4^] cage vary between ∼19% and ∼43% (Table S1), and their *U*_iso_ values were again fixed to 0.05 Å^2^. All C2 sites are within a spherically shaped area located between 1.24 and 2.30 Å off the virtual center of the [5^12^6^4^] cage (*i.e.* at *x* = 0.32, *y* = 0, *z* = 0.43) corresponding to distances ≥3.11 Å from the framework O atoms (Fig. 8 ▸). As a result of the partial site occupation, the ESDs of the positional parameters are high and thus a reliable assignment to individual mol­ecules is not possible from the obtained data. Fig. 8 ▸ provides a presentation of all possible C—C bonds in the range between 1.4 and 1.6 Å, which might be considered for the assignment of distinct hydro­carbon species. Difference Fourier summations of the LT data show high residual electron densities close to the O atoms monitoring displacive dislocation and unconsidered twin components.

### The RT model with space group *Fd* 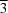 *m* and the LT structure evolution   

3.5.

The refinement of the crystal structure from the data set collected at RT (293 K) was performed in the space group 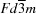, starting from the seven framework-atom positions reported by Könnecke *et al.* (1992 ▸). The refinement of the framework atoms with ADPs of all atoms converged at *R*_1_ = 0.083 and *wR*_2_ = 0.252. Residual electron densities up to 4.62 e Å^−3^ were located within the [5^12^] cages and up to 1.37 e Å^−3^ within the [5^12^6^4^] cages, with a distribution similar to that found in melanophlogite (Tribaudino *et al.*, 2008 ▸) and in the monoclinic LT structure. The C atoms were allocated to the centers of the residual electron densities. For their refinement, the SOFs were released and the *U*_iso_ values restrained to 0.05 Å^2^ according to the refinement of the monoclinic structure. The fully occupied C1 atom site was assigned to the center of the [5^12^] cage at *x* = 0, *y* = 0, *z* = 0. The maxima located within the larger [5^12^6^4^] cage centered at 3/8, 3/8, 3/8 were assigned to the five partially occupied C2 atom positions (C2a to C2e, expanded by the space-group symmetry to 44 positions) (Table S1). Again, too many tentative C—C bonds do not allow assignment of individual atoms to distinct hydro­carbon mol­ecules and hence do not provide clear evidence on individual alignments. The final refinement converged at *R*_1_ = 0.055 and *wR*_2_ = 0.133 for a total of 46 variable parameters including a scale factor and an extinction parameter (Table 1 ▸).

According to Momma *et al.* (2011 ▸), Na and Al are considered minor but essential constituents of chibaite. Based on electron-microprobe analyses, the authors gave the empirical formula for the host structure as Na_0.99_(Si_134.53_Al_1.63_)O_272_. This results in a moderate excess of cations; charge balance is achieved by Al^3+^ ions substituting the Si^4+^ ions. An additional weak maximum of the electron density (0.43 e Å^−3^) is located at 1/2, 1/2, 1/2, *i.e.* in the middle of the sixfold silicate rings linking the [5^12^6^4^] cages, which is ascribed to the small number of Na atoms (Fig. 8 ▸). A probable consequence of the lower resolution and some positional displacements was that the refinement of an analog position for Na atoms within the 100 K structure model was not possible. In zeolites, Na atoms are likely centered within sixfold silicate rings. The six Na—O1 bond lengths of 2.564 Å satisfy the crystal chemical requirements for charge balance. Moreover, the Si1—O1 bond length was found to be relatively long at 1.578 Å, and the Si1—O1—Si1 bond angle is slightly smaller compared with the two other angles at the bridging O atoms.

The X-ray diffraction images taken at RT exhibit an extremely slight increase in background in the regions where at LT the superstructure reflections are observed. Even though it was not possible to measure their intensities systematically, they indicate that the change of the structure type from 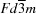 to *A*2/*n* symmetry already starts above RT. It is supported by the high and strongly anisotropic disk-shaped displacement parameters observed, especially for the atoms O2 and O4 at RT.

### The transformation path from space group *Fd* 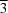 *m* to *A*2/*n*   

3.6.

Additional data sets were recorded in a series of different LT conditions, *i.e.* at 273, 250, 200 and 150 K. Although the data reduction based on cubic Laue symmetry is satisfactory for the measurements at *T* = 273 K, it yields increasingly strong misalignments with decreasing T as indicated by the increasing *R*_int_ values (Table 1 ▸). In addition, the intensities of the superstructure reflections become successively larger. For the data sets taken at *T* ≤ 250 K, the refinement in 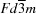 did not converge satisfactorily. The refinement of the C atom with the smallest SOF (C2a) did not converge and was therefore excluded. Likewise, it was not possible to refine the atomic coordinates of the atom C2b found in the 100 K data set. As dodecasil-3C was refined successfully in the space group 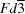 by Gies (1984 ▸), a similar approach was attempted. However, an analogous refinement of the chibaite structure using the data sets gathered at various T did not improve the results. Könnecke *et al.* (1992 ▸) discussed a model for calcined dodecasil-3C based on 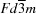 symmetry but with split positions for O2 and O3, as well as a release of the constraints of the atom O4. Accordingly, such a model was tested in this study. However, because of the extreme displacement of the O atoms occurring in a disk-shaped fashion, the refinement was not successful. The refinements in *A*2/*n* were possible only at *T* ≤ 250 K, with the reliability of the refinement increasing with decreasing T. At higher T, the superstructure reflections became too weak and could not be measured with sufficient significance. Since none of the cubic model variants converged for the measurements in the inter­mediate T range, and also since the refinements in *A*2/*n* did not result in a stable refinement with acceptable uncertainties, we refrain from presenting the results of these refinements. In our opinion, the results reflect that parts of the structure might be at least close to the 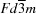 symmetry and other parts or co-existing domains in the crystal might exhibit the monoclinic *A*2/*n* symmetry. Concurrently, the degree of the deviation from cubic symmetry (for domains) appears to be the subject of change.

## 4. Discussion   

The single-crystal investigations of the naturally occurring sII-type hydro­carbon clathrasil, named chibaite, revealed a cubic 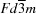 symmetry at RT in accordance with the topology of the MTN-type framework (as a gas hydrate, it is denoted by the sII structure). The lattice metrics provided no indication of a significant deviation from the cubic geometry. This finding is in agreement with the crystallographic data previously reported for dodecasil-3C, *i.e.* the synthetic analog of chibaite with an sII-type framework (Gies, 1984 ▸). Nevertheless, several symmetry variants for dodecasil-3C were observed under ambient conditions, depending on the type of guest mol­ecules (Gies, 1984 ▸; Chae *et al.* 1991 ▸; Könnecke *et al.*, 1992 ▸; Könnecke & Fuess, 1995 ▸; Knorr & Depmeier, 1997 ▸; Momma *et al.*, 2013 ▸; Momma, 2014 ▸).

During this study, a change in the cubic symmetry of chibaite with decreasing T was detected. Supported by the observed optical anisotropy and the formation of crystallographic domains in single crystals, the evolution of the Raman spectra and X-ray diffraction patterns imply a lowering of symmetry with decreasing T from 293 to 83 K. The measurements reported in this study reveal a significant change in symmetry from cubic to monoclinic. However, it is not possible to assign the structural changes to a distinct critical T as would be expected for distinct phase transitions. Moreover, the observed evolution down to 100 K does not allow for establishing a transition pathway from the cubic aristotype 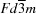 structure to the monoclinic subgroup *A*2/*n*. As a result, a transition from a point group of order 48 to one of order 4 is caused directly. For further investigations of the transition path from space group 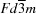 to *A*2/*n*, refinement of the measurements of the LT structures with synchrotron radiation would be worthwhile.

The silicate framework of the RT structure with the space group 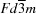 is topologically equivalent to that of the *A*2/*n* model at 100 K. One remarkable difference is the change in the Si—O bond lengths that were recalculated from the refined atomic coordinates determined by X-ray investigations. For the cubic RT structure, the Si—O values range between 1.536 and 1.583 Å with a mean value 〈Si—O〉 of 1.560 Å. Thus, they are shorter than those observed in the monoclinic LT structure, which range from 1.566 to 1.629 Å with mean 〈Si—O〉 values between 1.581 and 1.602 Å (Table 3*a* ▸). Furthermore, the bridging Si—O—Si angles are shallower in the cubic modification (169–180°) compared with the Si—O—Si angles of the monoclinic structure (149–177°; Figs. 7 ▸ and 8 ▸, Table 3*a* ▸). The Si—O bond lengths and Si—O—Si angles of the cubic modification deviate from the values of stable silicate framework structures. Instead, they show typical values for clathrasil structures, *e.g.* dodecasil-3C with 〈Si—O〉 = 1.566 Å and 〈Si—O—Si〉 = 174.5° (Gies, 1984 ▸), or melano­phlogite with 〈Si—O〉 = 1.578 Å and 〈Si—O—Si〉 = 16.3° (Tribaudino *et al.*, 2008 ▸). In chibaite, they approach the values for common silicates at 100 K, where the Si—O bond distances average around 1.608 Å (Brown & Gibbs, 1969 ▸; Brown *et al.*, 1969 ▸; Liebau, 1985 ▸) and the Si—O—Si angles around 144° (Tossell & Gibbs, 1978 ▸). Brown & Gibbs (1969 ▸), Brown *et al.* (1969 ▸) and Tribaudino *et al.* (2008 ▸) reported the relationship between Si—O bond lengths and Si—O—Si angles, concluding that large Si—O—Si angles correlate with small Si—O bond lengths. The short bond distances associated with straight or near-straight bridging angles between SiO_4_ units of the RT chibaite indicate a high degree of displacement in a static and/or dynamic fashion. In particular, the bridging O atoms show large displacements exhibiting disk-like shapes of their ADPs. In both structures, the Si atom positions exhibit only a moderate mean displacement, whereas the positional shifts of the O atoms are rather pronounced. Their ADPs are large for the LT structure but display even higher values, along with a drastically higher anisotropy, at RT (Table S1). Consequently, the dynamic or even static displacement in the RT framework around the O atom barycenters is larger than in the LT phase. The structural changes are likely to originate from the instability of the cubic host framework at RT. The comparison of both structures is shown in Figs. 7 ▸ and 8 ▸. The transformation might also be triggered by the ordering and alignment of the guest mol­ecules. Apart from CH_4_, CO_2_ and N_2_, which probably exclusively occupy the smaller [5^12^] cage type, other hydro­carbon mol­ecules (*i.e.* C_2_H_6_, C_3_H_8_, *i*-C_4_H_10_) are distributed between both cage types, *i.e.* [5^12^] and [5^12^6^4^], as derived from the Raman spectra in this study. A limiting factor for the occupation of the cages is their size. In the cubic phase, the diameters are 8.3 ± 3 Å for [5^12^] and 9.9 ± 4 Å for [5^12^6^4^], *i.e.* twice the value of the 〈*i*—O〉 distances, where *i* is the respective cage center (Table 3*b* ▸). Considering the ionic radius of O^[2]^ atoms (1.35 Å) (Shannon, 1976 ▸), the effective diameters are ∼5.6 and ∼7.4 Å on average in the 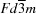 structure. The symmetry reduction resulting from the 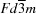 to *A*2/*n* transformation leads to a significant increase in distortion and, consequently, a larger range for individual *i*—O distances of the four individual [5^12^] cages (*i.e.* 3.6–4.7 Å), even if the average 〈C—O〉 bond lengths are consistent (4.13 Å in 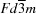 and 4.14–4.16 Å in *A*2/*n*). In contrast, the unique [5^12^6^4^] cage type remains rather regular (*i*—O range from 4.9 to 5.1 Å); the effective pore sizes range from 4.5 to 6.7 Å and from 7.1 to 7.4 Å for the [5^12^] and [5^12^6^4^] cages, respectively. With respect to the kinetic diameters of the hydro­carbons (3.8, 3.8, 4.3 and 5.0 Å; Breck, 1974 ▸) the distribution of the various hydro­carbon types seems possible. Even the largest hydro­carbon mol­ecule *i*-C_4_H_10_, with its diameter of 5.0 Å, is still compatible with a location along the largest diameter of 6.7 Å inside the [5^12^] cage at LT. The formally calculated largest effective diameter in the RT structure with the space group 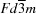 for the [5^12^] cage is only ∼5.6 Å, and thus barely suitable for the larger hydro­carbons. However, the ADPs of the O atoms are up to 0.17 Å^2^. Thus, the distribution of the various hydro­carbon types is compatible with both cages at RT as well as LT.

The assignment of distinct alignment positions of the mol­ecules is not feasible because of the partial occupation and relatively high degree of freedom of displacement, resulting in large displacement parameters. Only the C1 positions in the centers of the [5^12^] cages are (almost) completely occupied over the whole T range investigated. However, the large correlation between the SOFs and the displacement parameters does not allow for a detailed allocation. The sites are assumed to be the barycenters of the CH_4_, CO_2_, C_3_H_8_, C_2_H_6_ or *i-*C_4_H_10_ mol­ecules. The positions located within the larger [5^12^6^4^] cage exhibit much weaker electron densities as a result of extensive disorder. Therefore, they reveal only partial site occupancies and their assignment is not possible. For the structure model in space group 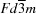, the located electron-density maxima and refined atomic sites are most likely a result of the varied occupation of symmetrically equivalent positions in distinct host-framework cages. It should be mentioned that most of the partially occupied positions within the [5^12^6^4^] cage are arranged close to a centered sphere with a radius of approximately 1.4 Å; this causes distances to neighboring O atoms of about 3 Å, which is in accordance with the expected values for C—H⋯O hydrogen bonds. However, in the space group *A*2/*n*, the maxima are unique and therefore their number is smaller. A high degree of dynamic disorder with respect to mol­ecule orientation is assumed.

## 5. Conclusions   

Raman and sXRD investigations of chibaite at RT and at various LTs down to 100 K revealed a continuous phase transformation over the investigated T range. In accordance with earlier studies, chibaite crystallizes in the space group 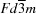 at RT, the structure type of dodecasil-3C, consisting of an SiO_2_ host with mainly hydro­carbon guest mol­ecules. Extremely disk-shaped ADPs, especially for the O atoms, suggest a static or dynamic disorder and might indicate a structural instability. As T decreases, the high symmetry continuously decreases over a certain T range. A distinct T of the phase transition cannot be verified. Reconstructed X-ray diffraction patterns reveal weak superstructure reflections whose intensities increase with decreasing T. The transformation according to the transformation matrix 
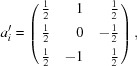
finally results in a monoclinic phase with *A*2/*n* symmetry, which is twinned according to the twin law
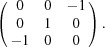
Both cells with space groups 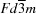 and *A*2/*n* have analogous cell volumes. In *A*2/*n*, the host framework has Si—O bond lengths and Si—O—Si angles that are much closer to the values known for stable silicate-framework structures compared with the RT structure with the space group 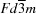 model. Surprisingly, band splitting in the Raman spectra suggests that the hydro­carbon guest mol­ecules C_2_H_6_, C_3_H_8_ and *i*-C_4_H_10_ occupy both cages. The larger [5^12^6^4^]-type cage was found to be unique in both structure types. The [5^12^]-type cages (one crystallographically unique in 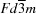, four different in *A*2/*n*) entrap the hydro­carbons CH_4_, C_2_H_6_, C_3_H_8_ and *i*-C_4_H_10_. Small amounts of Na atoms are located in the centers of the six-membered rings which constitute the cage walls of the host.

## Supplementary Material

Crystal structure: contains datablock(s) 293k, 273k, 250k_mcl, 250k_cubic, 200k_mcl, 200k_cubic, 150k_mcl, 150k_cubic, 100k_mcl, 100k_cubic. DOI: 10.1107/S2052252518009107/lt5009sup1.cif

Structure factors: contains datablock(s) 293k. DOI: 10.1107/S2052252518009107/lt5009293ksup2.hkl

Structure factors: contains datablock(s) 273k. DOI: 10.1107/S2052252518009107/lt5009273ksup3.hkl

Structure factors: contains datablock(s) 250k_mcl. DOI: 10.1107/S2052252518009107/lt5009250k_mclsup4.hkl

Structure factors: contains datablock(s) 250k_cubic. DOI: 10.1107/S2052252518009107/lt5009250k_cubicsup5.hkl

Structure factors: contains datablock(s) 200k_mcl. DOI: 10.1107/S2052252518009107/lt5009200k_mclsup6.hkl

Structure factors: contains datablock(s) 200k_cubic. DOI: 10.1107/S2052252518009107/lt5009200k_cubicsup7.hkl

Structure factors: contains datablock(s) 150k_mcl. DOI: 10.1107/S2052252518009107/lt5009150k_mclsup8.hkl

Structure factors: contains datablock(s) 150k_cubic. DOI: 10.1107/S2052252518009107/lt5009150k_cubicsup9.hkl

Structure factors: contains datablock(s) 100k_mcl. DOI: 10.1107/S2052252518009107/lt5009100k_mclsup10.hkl

Structure factors: contains datablock(s) 100k_cubic. DOI: 10.1107/S2052252518009107/lt5009100k_cubicsup11.hkl

Supplementary table. DOI: 10.1107/S2052252518009107/lt5009sup12.pdf

CCDC references: 1854241, 1854242, 1854243, 1854244, 1854245, 1854246, 1854247, 1854248, 1854249, 1854250

## Figures and Tables

**Figure 6:**
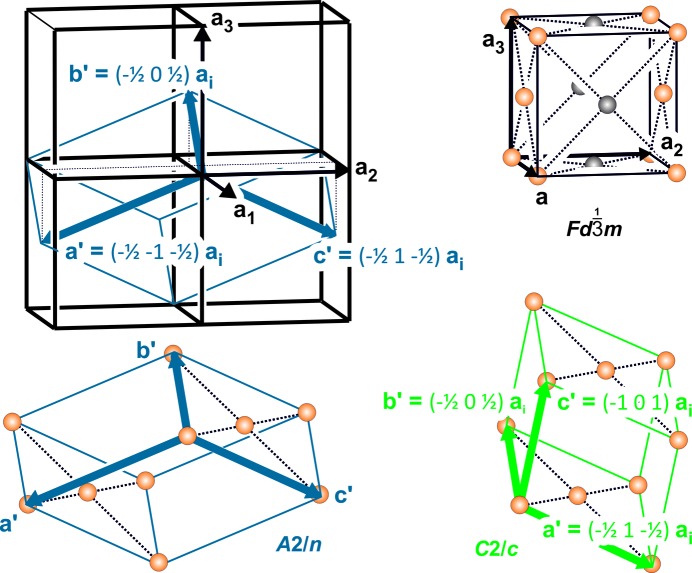
Relationship between the 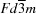 unit cell (*a* ≃ 19.4 Å, displayed in black) and the monoclinic *A*2/*n* cell (*a*′ = *c*′ ≃ 23.7 Å and *b*′ ≃ 13.7 Å, in blue; analogous *C*2/*c* setting in green). The identical lattice points of the cubic *F* lattice and the monoclinic *A* lattice are marked by yellow spheres. Gray spheres in the 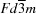 cell lose translation identity in *A*2/*n* and *C*2/*c*.

**Table 1:** X-ray intensity data collections and single-crystal structure refinements of chibaite in the temperature range from 293 to 100 K Refinements were carried out in the space groups 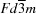 (No. 227) and *A*2/*n* (No. 15); β_monoclinic_ = 109.47°, *a*_mon_ = *c*_mon_ = (a_cub_/2) × 2^1/2^ × 3^1/2^, *b*_mon_ = (*a*_cub_/2) × 2^1/2^, β = 2arctan 2^1/2^ = 109.47°, *Z*_cub_ = *Z*_mon_ = 136 [SiO_2_·*x* (CH_4_, C_2_H_6_, C_3_H_8_, *i*-C_4_H_10_, CO_2_, Na)].

Temperature (K)	293		273		250		200		150		100	
*a*_cubic_ (Å)	19.4420 (15)		19.4214 (15)		19.4199 (15)		19.3944 (15)		19.3944 (15)		19.3944 (15)	
*V*_cubic_ (Å^3^)	7348.9 (17)		7325.6 (17)		7323.9 (17)		7295.1 (17)		7295.1 (17)		7295.1 (17)	
*a*_monoclinic_ (Å)	23.812 (2)		23.786 (2)		23.7844 (3)		23.834 (5)		23.834 (3)		23.7054 (2)	
*b*_monoclinic_ (Å)	13.7476 (11)		13.7330 (11)		13.732 (5)		13.760 (5)		13.760 (5)		13.6861 (11)	
*c*_monoclinic_ (Å)	23.812 (2)		23.786 (2)		23.784 (3)		23.833 (3)		23.833 (3)		23.7051 (2)	
Scan time (s)/width (°)	120/0.5		40/0.5		40/0.5		40/0.5		40/0.5		40/0.5	
Collected frames	910		1408		1408		1408		1408		1408	
Space group symmetry	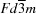	*A*2/*n*	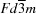	*A*2/*n*	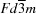	*A*2/*n*	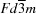	*A*2/*n*	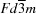	*A*2/*n*	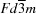	*A*2/*n*
Measured reflections	30717	–	30687	–	30685	84218	30560	92816	30481	92644	30099	93896
Unique reflections	721	–	722	–	722	10601	720	12998	721	12943	718	12953
Reflections |*F*_o_| > 4σ	591	–	638	–	688	4856	699	6288	695	7599	695	7878
*R*_int_ †	0.096	–	0.106	–	0.21	0.162	0.254	0.085	0.303	0.113	0.33	0.138
*R*_1_ ‡ (|*F*_o_| > 4σ)	0.055	–	0.080	–	0.167	0.21	0.20	0.18	0.22	0.116	0.23	0.120
*R*_1_ ‡ (all data)	0.067	–	0.088	–	0.172	0.34	0.20	0.30	0.23	0.173	0.24	0.172
*wR*_2_ § (all data)	0.133	–	0.16	–	0.32	0.280	0.38	0.278	0.415	0.186	0.43	0.199
GooF¶	1.11	–	1.26	–	1.28	3.11	1.29	2.91	1.20	1.98	1.14	2.00
Variable parameters	46	–	46	–	44	210	44	214	44	533	50	533
Extinction	0.00036 (9)	–	0.00035 (9)	–	0.0009 (3)	n.d.	0.0013 (3)	n.d.	0.0017 (4)	n.d.	0.0039 (7)	n.d.
Electron density min, max (e Å^−3^)	−1.24 1.01	–	−1.26, 0.97	–	−5.98, 1.21	−2.90, 10.05	−6.63, 1.43	−2.65, 6.88	−7.50, 1.57	−2.23, 2.18	−5.89, 1.50	−2.16, 2.19
Violations *d*-glide plane	0	–	18	–	323	–	472	–	556	–	585	–
Twin fraction (only in *A*2/*n*)	–	–	–	–	–	0.674 (4)	–	0.518 (4)	–	0.165 (2)	–	0.155 (2)

† *R*_int_ = 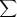
∣*F*_o_^2^−*F*_o_^2^(mean)∣/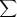 *F*_o_^2^.

‡ *R*_1_ = 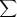
(∣∣*F*_o_∣-∣*F*_c_∣∣)/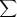 *F*_o_.

§ *wR*_2_ = [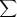 *w*(*F*_o_^2^−*F*_c_^2^)^2^/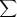
∣*wF*_o_^4^]^1/2^.

¶ GooF = {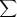
[*w*(*F*_o_^2^−*F*_c_^2^)^2^]/(*n*−*p*)}^0.5^.

**Table 2:** Individual Raman vibrations of the guest mol­ecules and their assignment to the two types of framework cages at *T* = 293 K

Guest mol­ecule	Vibration	Cage type	*ν*_guest_ (cm^−1^)
Isobutane, *i*-C_4_H_10_ (Klapp *et al.*, 2010 ▸)	C—C symmetric stretching	[5^12^]	804.8 (5)
		[5^12^6^4^]	811.6 (5)
Propane, C_3_H_8_ (Sum *et al.*, 1997 ▸)	C—C symmetric stretching	[5^12^]	871.8 (5)
		[5^12^6^4^]	891.2 (5)
Ethane, C_2_H_6_ (Klapp *et al.*, 2010 ▸)	C—C symmetric stretching (*ν*_3_)	[5^12^]	988.9 (5)
		[5^12^6^4^]	997.8 (5)
Methane, CH_4_ (Sum *et al.*, 1997 ▸)	C—H symmetric stretching (*ν*_1_)	[5^12^]	2907.5 (5)
	Overtone of C—H asymmetric bending vibration (2*ν*_2_)	[5^12^]	3049.8 (5)
N_2_ (Tribaudino *et al.*, 2008 ▸)		[5^12^]	2322.3 (5)
CO_2_ (Charlou *et al.*, 2004 ▸)	Fermi dyad (*ν*_c−_) and (*ν*_c+_)	[5^12^]	1271.9 (5)
			1380.0 (5)

**Table:** Tentative C—C bond distances are given in the range 1.40–1.60 Å. At 100 K, variation of the individual and the average bond distances and angles are given as ranges with the minimum and maximum values for the crystallographically independent 12 Si1*x*O_4_, four Si2*x*O_4_ and the one Si3O_4_ unit. The variation of the individual and average values corresponds to the range for 13 O1*x*, 12 O2*x*, 6 O3*x* and 4 O4*x* atoms.(*a*) 293 K (space group 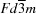)[5^12^]-cage center corresponds to C1; [5^12^6^4^]-cage center located at 8*b* (= 3/8 3/8 3/8) with site symmetry 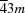.

Framework atoms				
Si1—O2	1.569 (4)		O—Si1—O	108.7 (3)–110.3 (3)
Si1—O1	1.5780 (8)	2×	O—Si2—O	108.4 (2)–110.6 (2)
Si1—O3	1.5831 (11)		O—Si3—O	109.47
Si2—O4	1.536 (7)		Si1—O1—Si1	168.8 (4)
Si2—O2	1.555 (4)	3×	Si1—O2—Si2	179.2 (5)
Si3—O4	1.538 (7)	4×	Si1—O3—Si1	174.9 (4)
〈Si—O〉	1.560		〈Si—O—Si〉	174.3
Na—O1	2.564 (5)	6×	Si2—O4—Si3	180

**Table:** 

Extra-framework atoms				
C1—O	3.971 to 4.313		8(b)—O	4.893 to 5.067
〈C1^[30]^—O〉	>4.133		〈8(b)^[42]^—O〉	>4.962
				
C2a—C2d	1.51 (7)		C2d—C2e	1.51 (8)
C2b—C2e	1.48 (6)		C2d—C2a	1.51 (7)
C2b—C2d	1.58 (7)		C2d—C2b	1.58 (7)
C2c—C2e	1.44 (4)		C2e—C2c	1.43 (4)
C2c—C2d	1.49 (3)		C2e—C2b	1.48 (6)
C2c—C2e	1.55 (8)		C2e—C2d	1.51 (8)
			C2e—C2c	1.55 (8)

**Table:** (*b*) 100 K (space group *A*2/*n*)[5^12^]-cage center corresponds to C1a, C1b, C1c, C1d; [5^12^6^4^]-cage center located at position 8(*f*) (0.23 0.00 0.43), site symmetry 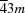

Framework atoms			
Si1*x*—O*x*	1.566 (10)–1.629 (9)	〈Si1*x*—O*x*〉	1.581–1.601
Si2*x*—O*x*	1.568 (11)–1.596 (10)	〈Si2*x*—O*x*〉	1.582–1.584
Si3*x*—O*x*	1.569 (11)–1.612 (8)	〈Si3—O4〉	1.588
O*x*—Si1*x*—O*x*	105.5 (5)–112.1 (5)	〈O*x*—Si1*x*—O*x*〉	109.43–109.47
O*x*—Si2*x*—O*x*	106.3 (7)–112.2 (5)	〈O*x*—Si2*x*—O*x*〉	109.45–109.46
O4*x*—Si3—O4*x*	107.4 (6)–111.1 (6)	〈O4*x*—Si3—O4*x*〉	109.46
Si1*x*—O1*x*—Si1*x*	149.3 (6)–173.6 (9)	〈Si1*x*—O1*x*—Si1*x*〉	160.8
Si1*x*—O2*x*—Si2*x*	151.2 (7)–168.5 (9)	〈Si1*x*—O2*x*—Si2*x*〉	156.9
Si1*x*—O3*x*—Si1*x*	161.7 (8)–176.5 (3)	〈Si1*x*—O3*x*—Si1*x*〉	171.3
Si2*x*—O4*x*—Si3	146.8 (5)–156.5 (9)	〈Si2*x*—O4*x*—Si3〉	151.2

**Table:** 

Extra-framework atoms			
C1a—O	3.584–4.650	〈C1a^[30]^—O〉	>4.141
C1c—O	3.665–4.516	〈C1c^[30]^—O〉	>4.152
C1b—O	3.630–4.684	〈C1b^[30]^—O〉	>4.133
C1d—O	3.632–4.703	〈C1d^[30]^—O〉	>4.135
8(*f*)—O	4.554–5.470	〈8(*f*)^[42]^—O〉	>4.970
C2b—C2c	1.41 (8)	C2d—C2i	1.49 (9)
C2c—C2j	1.58 (9)	C2g—C2i	1.47 (9)
