# Supplementary material for: The hydrocarbon-bearing clathrasil chibaite and its host–guest structure at low temperature
Source: IUCrJ. 2018 Aug 8;5(Pt 5):595–607. doi: 10.1107/S2052252518009107 (PMC6126654; doi:10.1107/S2052252518009107)
Supplement: Supplementary file 12 [file m-05-00595-sup12.pdf]

IUCrJ

Volume 5 (2018)

Supporting information for article:

The hydrocarbon-bearing clathrasil chibaite and its host–guest structure at low temperature

K. S. Scheidl, H. S. Effenberger, T. Yagi, K. Momma and Ronald Miletich

Table S1: Fractional atomic coordinates and displacement parameters of chibaite at 293 K (space group $Fd\bar{3}m$) and 100 K (space group $A2/n$). The anisotropic displacement parameters are defined as: $\exp[-2\pi^2 \Sigma^3_{i=1} \Sigma^3_{j=1} U_{ij}\, a^*_i\, a^*_j\, h_i\, h_j]$. C atoms located in the [5^{12}] and [$5^{12}6^6$] cage are labelled C1 and C1x, respectively C2x (x = a,b,c….,l).

Space group $Fd\bar{3}m$, 293 K, origin choice 2, origin at centre $\bar{3}m$ at 1/8 1/8 1/8 from $\bar{4}3m$

Atom	SOF	Wyckoff position		x/a	y/b	z/c	U_{equiv} / U_{iso}	U_{11}	U_{22}	U_{33}	U_{23}	U_{13}	U_{12}
**Si1**	1	96(*g*)	..*m*	0.06748(4)	= *x*	0.36973(5)	0.0244(3)	0.0261(4)	= U_{11}	0.0210(5)	-0.0049(3)	= U_{23}	0.0036(3)
**Si2**	1	32(*e*)	.3*m*	0.21629(5)	= *x*	= *x*	0.0180(4)	0.0180(4)	= U_{11}	= U_{11}	-0.0012(3)	= U_{23}	= U_{23}
**Si3**	1	8(*a*)	$\bar{4}3m$	1/8	1/8	1/8	0.0181(6)	0.0181(6)	= U_{11}	= U_{11}	0	0	0
**O1**	1	96(*h*)	..2	0	0.3433(2)	= -*y*	0.0627(12)	0.034(2)	0.077(2)	= U_{22}	-0.017(2)	-0.0122(12)	= U_{12}
**O2**	1	96(*g*)	..*m*	0.1999(2)	= x	0.2928(2)	0.083(2)	0.108(3)	= U_{11}	0.032(2)	0.027(2)	= U_{23}	0.004(3)
**O3**	1	48(*f*)	2.*mm*	0.3734(3)	1/8	1/8	0.0514(13)	0.063(4)	0.046(2)	= U_{22}	-0.014(2)	0	0
**O4**	1	32(*e*)	.3*m*	0.1707(2)	= *x*	= *x*	0.120(4)	0.120(4)	= U_{11}	= U_{11}	-0.054(3)	= U_{23}	= U_{23}
**Na**	0.031(14)	16(*d*)	$.\bar{3}m$	1/2	1/2	1/2	0.05[a]						
**C1**	1	16(*c*)	$.\bar{3}m$	0	0	0	0.083(6)	0.083(6)	= U_{11}	= U_{11}	-0.010(6)	= U_{23}	= U_{23}
**C2a**	0.10(5)	32(*e*)	.3*m*	0.332(4)	= *x*	= *x*	0.05[a]						
**C2b**	0.11(2)	32(*e*)	.3*m*	0.392(3)	= *x*	= *x*	0.05[a]						
**C2c**	0.154(14)	96(*g*)	..*m*	0.3614(13)	= *x*	0.302(2)	0.05[a]						
**C2d**	0.113(13)	96(*g*)	..*m*	0.343(2)	= *x*	0.434(2)	0.05[a]						
**C2e**	0.117(19)	96(*g*)	..*m*	0.324(2)	= *x*	0.361(4)	0.05[a]						

Space group *A*2/*n*, 100 K

**Atom**	**SOF**	**Wyckoff position**		***x/a***	***y/b***	***z/c***	U_{equiv} / U_{iso}	U_{11}	U_{22}	U_{33}	U_{23}	U_{13}	U_{12}
**Si1a**	1	8(*f*)	1	0.01340(10)	0.9996(2)	0.14474(9)	0.0073(4)	0.0143(9)	0.0062(8)	0.0043(8)	0.0001(8)	0.0068(7)	-0.0003(9)
**Si1b**	1	8(*f*)	1	0.18513(12)	0.2006(2)	0.25464(11)	0.0108(5)	0.0119(12)	0.0128(11)	0.0100(10)	0.0019(8)	0.0065(9)	0.0010(9)
**Si1c**	1	8(*f*)	1	0.18563(12)	0.7878(2)	0.25689(11)	0.0100(5)	0.0100(12)	0.0152(11)	0.0066(10)	-0.0029(8)	0.0051(9)	-0.0021(9)
**Si1d**	1	8(*f*)	1	0.99731(12)	0.3127(2)	0.43511(11)	0.0111(5)	0.0159(13)	0.0098(11)	0.0073(10)	0.0022(8)	0.0033(9)	-0.0038(9)
**Si1e**	1	8(*f*)	1	0.99725(12)	0.6910(2)	0.43338(11)	0.0107(5)	0.0151(13)	0.0080(10)	0.0081(10)	-0.0038(8)	0.0026(9)	0.0018(9)
**Si1f**	1	8(*f*)	1	0.18483(12)	0.8862(2)	0.57046(12)	0.0113(5)	0.0109(12)	0.0119(11)	0.0112(11)	0.0014(8)	0.0040(9)	0.0004(9)
**Si1g**	1	8(*f*)	1	0.18673(12)	0.1158(2)	0.56471(11)	0.0117(5)	0.0127(12)	0.0132(11)	0.0097(11)	-0.0024(8)	0.0043(9)	0.0000(9)
**Si1h**	1	8(*f*)	1	0.18316(12)	0.3131(2)	0.37075(12)	0.0115(5)	0.0119(12)	0.0067(10)	0.0185(12)	0.0001(9)	0.0083(10)	0.0001(9)
**Si1i**	1	8(*f*)	1	0.18242(12)	0.6892(2)	0.37713(12)	0.0116(5)	0.0131(12)	0.0078(10)	0.0158(11)	0.0023(8)	0.0073(10)	-0.0005(9)
**Si1j**	1	8(*f*)	1	0.13172(10)	0.9953(2)	0.25949(10)	0.0106(4)	0.0142(9)	0.0103(10)	0.0103(9)	-0.0003(9)	0.0078(7)	-0.0006(10
**Si1k**	1	8(*f*)	1	0.11114(13)	0.8166(2)	0.43997(12)	0.0121(5)	0.0143(12)	0.0140(11)	0.0096(10)	0.0029(9)	0.0060(9)	0.0025(9)
**Si1l**	1	8(*f*)	1	0.11091(12)	0.1929(2)	0.43566(11)	0.0112(5)	0.0146(12)	0.0123(11)	0.0081(10)	-0.0032(8)	0.0057(9)	-0.0050(9)
**Si2a**	1	8(*f*)	1	0.89253(9)	0.9966(2)	0.17178(10)	0.0089(4)	0.0076(8)	0.0097(9)	0.0104(9)	0.0028(9)	0.0044(7)	-0.0007(9)
**Si2b**	1	8(*f*)	1	0.10915(13)	0.6782(2)	0.14063(12)	0.0111(5)	0.0133(13)	0.0097(11)	0.0103(11)	0.0004(8)	0.0039(10)	-0.0012(9)
**Si2c**	1	8(*f*)	1	0.89373(13)	0.1842(2)	0.35530(13)	0.0132(5)	0.0135(13)	0.0104(11)	0.0151(12)	-0.0015(9)	0.0039(10)	-0.0001(9)
**Si2d**	1	8(*f*)	1	0.07440(10)	0.0018(2)	0.35978(9)	0.0096(4)	0.0144(9)	0.0093(9)	0.0058(8)	0.0000(9)	0.0045(7)	0.0013(10)
**Si3**	1	8(*f*)	1	0.93748(11)	0.9990(2)	0.30819(10)	0.0115(4)	0.0167(10)	0.0105(9)	0.0092(9)	-0.0008(9)	0.0069(7)	-0.0033(11)
**O1a**	1	4(*e*)	2	1/4	0.2181(9)	1/4	0.027(3)	0.012(5)	0.025(6)	0.053(8)	0	0.022(6)	0
**O1b**	1	4(*e*)	2	1/4	0.7764(9)	1/4	0.025(2)	0.020(6)	0.026(6)	0.037(6)	0	0.022(5)	0
**O1c**	1	8(*f*)	1	0.0119(5)	0.9034(7)	0.1078(4)	0.038(2)	0.064(7)	0.022(4)	0.023(4)	-0.016(3)	0.008(4)	0.004(4)
**O1d**	1	8(*f*)	1	0.2499(4)	0.8392(8)	0.5904(5)	0.041(2)	0.019(4)	0.036(5)	0.061(7)	0.002(5)	0.006(5)	0.014(4)
**O1e**	1	8(*f*)	1	0.9994(4)	0.3416(6)	0.5012(4)	0.0239(14)	0.037(4)	0.022(3)	0.013(3)	0.003(3)	0.009(3)	0.007(4)
**O1f**	1	8(*f*)	1	0.3561(5)	0.6538(9)	0.4994(4)	0.039(2)	0.040(6)	0.053(7)	0.020(4)	-0.018(4)	0.004(4)	-0.005(5)
**O1g**	1	8(*f*)	1	0.1461(5)	0.8354(8)	0.5098(5)	0.041(3)	0.050(7)	0.040(6)	0.030(5)	-0.011(4)	0.007(5)	-0.009(5)
**O1h**	1	8(*f*)	1	0.1578(5)	0.2456(8)	0.4110(4)	0.034(2)	0.036(5)	0.042(5)	0.034(5)	0.005(4)	0.024(4)	-0.010(4)
**O1i**	1	8(*f*)	1	0.1713(4)	0.8999(6)	0.2591(4)	0.027(2)	0.030(5)	0.019(4)	0.030(4)	-0.001(3)	0.007(4)	0.011(3)
**O1j**	1	8(*f*)	1	0.1565(4)	0.7679(7)	0.4121(4)	0.030(2)	0.029(5)	0.034(5)	0.035(5)	-0.006(4)	0.021(4)	0.005(4)

**O1k**	1	8(*f*)	1	0.0164(5)	0.0917(6)	0.1056(4)	0.032(2)	0.054(6)	0.021(4)	0.021(4)	0.015(3)	0.011(4)	-0.006(4)
**O1l**	1	8(*f*)	1	0.2520(4)	0.1617(7)	0.5779(4)	0.029(2)	0.026(4)	0.032(5)	0.033(5)	-0.006(4)	0.014(4)	-0.010(4)
**O1m**	1	8(*f*)	1	0.1740(4)	0.0869(6)	0.2602(4)	0.029(2)	0.028(5)	0.017(4)	0.043(5)	0.001(3)	0.012(4)	-0.007(3)
**O2a**	1	8(*f*)	1	0.1346(5)	0.7363(7)	0.2017(4)	0.033(2)	0.036(5)	0.039(5)	0.027(5)	-0.015(4)	0.015(4)	-0.006(4)
**O2b**	1	8(*f*)	1	0.8650(5)	0.2619(10)	0.3051(5)	0.052(3)	0.028(5)	0.074(9)	0.044(6)	0.039(6)	-0.002(5)	0.001(5)
**O2c**	1	8(*f*)	1	0.8606(5)	0.9030(8)	0.1346(5)	0.041(3)	0.052(7)	0.028(5)	0.043(6)	-0.004(4)	0.017(5)	-0.021(5)
**O2d**	1	8(*f*)	1	0.0592(5)	0.7385(9)	0.0917(5)	0.045(3)	0.037(6)	0.049(7)	0.040(6)	0.007(5)	0.003(5)	0.024(5)
**O2e**	1	8(*f*)	1	0.9443(5)	0.2362(8)	0.4090(4)	0.041(3)	0.046(7)	0.045(6)	0.024(5)	0.004(4)	0.000(4)	-0.031(5)
**O2f**	1	8(*f*)	1	0.0802(6)	0.1027(8)	0.3940(5)	0.048(3)	0.059(7)	0.037(6)	0.059(7)	-0.037(5)	0.034(6)	-0.023(5)
**O2g**	1	8(*f*)	1	0.8453(5)	0.1333(8)	0.3781(5)	0.039(3)	0.044(6)	0.046(6)	0.034(5)	-0.010(4)	0.023(5)	-0.028(5)
**O2h**	1	8(*f*)	1	0.1173(4)	0.9958(8)	0.3210(4)	0.032(2)	0.028(4)	0.052(6)	0.017(3)	0.005(4)	0.010(3)	0.004(5)
**O2i**	1	8(*f*)	1	0.0874(6)	0.9183(8)	0.4083(5)	0.048(3)	0.073(8)	0.042(6)	0.046(6)	0.033(5)	0.041(6)	0.032(6)
**O2j**	1	8(*f*)	1	0.1614(5)	0.6529(9)	0.1148(4)	0.040(3)	0.030(5)	0.063(7)	0.029(5)	-0.015(5)	0.014(4)	0.007(5)
**O2k**	1	8(*f*)	1	0.8565(6)	0.0914(9)	0.1447(6)	0.059(4)	0.075(10)	0.038(7)	0.064(8)	0.008(6)	0.023(7)	0.040(6)
**O2l**	1	8(*f*)	1	0.9563(4)	0.0063(9)	0.1653(4)	0.036(2)	0.021(3)	0.066(7)	0.030(4)	-0.011(5)	0.019(3)	-0.010(5)
**O3a**	1	8(*f*)	1	0.0717(3)	0.9979(7)	0.2037(3)	0.0247(14)	0.024(3)	0.025(4)	0.020(3)	0.002(3)	0.001(3)	-0.002(4)
**O3b**	1	8(*f*)	1	0.1898(4)	0.0001(7)	0.5631(4)	0.030(2)	0.036(4)	0.017(3)	0.040(5)	0.006(4)	0.016(3)	0.001(4)
**O3c**	1	8(*f*)	1	0.9403(5)	0.2310(8)	0.0627(5)	0.036(2)	0.031(5)	0.044(6)	0.037(5)	0.004(4)	0.016(4)	0.015(4)
**O3d**	1	8(*f*)	1	0.0573(5)	0.7470(9)	0.4351(5)	0.045(3)	0.033(6)	0.063(8)	0.047(6)	-0.019(5)	0.024(5)	-0.028(5)
**O3e**	1	8(*f*)	1	0.1854(4)	0.7345(7)	0.3171(4)	0.028(2)	0.037(5)	0.033(5)	0.024(4)	-0.003(3)	0.022(4)	-0.002(4)
**O3f**	1	8(*f*)	1	0.1813(5)	0.2571(8)	0.3121(5)	0.039(3)	0.054(7)	0.036(5)	0.041(6)	-0.011(4)	0.034(5)	-0.009(5)
**O4a**	1	8(*f*)	1	0.0075(3)	0.9923(8)	0.3159(3)	0.028(2)	0.017(3)	0.050(5)	0.017(3)	-0.006(4)	0.007(3)	-0.004(4)
**O4b**	1	8(*f*)	1	0.9257(7)	0.1038(9)	0.3286(8)	0.064(4)	0.069(9)	0.026(5)	0.120(13)	-0.019(7)	0.060(9)	0.007(6)
**O4c**	1	8(*f*)	1	0.8988(4)	0.9841(10)	0.2401(4)	0.042(3)	0.023(4)	0.089(9)	0.016(4)	0.004(5)	0.008(3)	-0.007(5)
**O4d**	1	8(*f*)	1	0.9216(6)	0.9189(8)	0.3492(4)	0.050(3)	0.082(9)	0.046(6)	0.025(5)	0.003(4)	0.020(5)	-0.039(6)
**C1a**	1	4(*c*)	$\bar{1}$	0	1/4	1/4	0.034(4)	0.043(12)	0.031(10)	0.030(9)	0.005(7)	0.014(9)	-0.007(8)
**C1b**	1	4(d)	$\bar{1}$	0	1/4	3/4	0.042(6)	0.07(2)	0.024(9)	0.055(14)	0.008(9)	0.043(14)	0.021(10)
**C1c**	1	4(*e*)	2	3/4	0.001(2)	1/4	0.033(3)	0.035(8)	0.040(9)	0.019(7)	0	0.003(6)	0
**C1d**	1	4(*b*)	$\bar{1}$	0	0	1/2	0.036(4)	0.051(10)	0.045(10)	0.022(7)	-0.013(8)	0.027(7)	-0.010(10)
**C2a**	0.43(4)	8(*f*)	1	0.817(2)	0.412(3)	0.409(2)	0.05[a]						

**C2b**	0.35(5)	8(*f*)	1	0.297(3)	0.905(4)	0.402(2)	0.05[a]
**C2c**	0.35(4)	8(*f*)	1	0.827(3)	0.092(4)	-0.036(2)	0.05[a]
**C2d**	0.26(5)	8(*f*)	1	0.273(3)	0.078(5)	0.430(3)	0.05[a]
**C2e**	0.39(5)	8(*f*)	1	0.189(2)	0.018(4)	0.124(2)	0.05[a]
**C2f**	0.31(5)	8(*f*)	1	0.183(3)	0.947(5)	0.115(3)	0.05[a]
**C2g**	0.26(4)	8(*f*)	1	0.175(3)	0.587(6)	0.532(3)	0.05[a]
**C2h**	0.21(5)	8(*f*)	1	0.255(4)	0.014(7)	0.457(4)	0.05[a]
**C2i**	0.35(5)	8(*f*)	1	0.291(2)	0.001(4)	0.478(2)	0.05[a]
**C2j**	0.23(5)	8(*f*)	1	0.277(4)	0.935(6)	0.456(4)	0.05[a]
**C2k**	0.26(5)	8(*f*)	1	0.871(3)	0.498(6)	0.462(3)	0.05[a]
**C2l**	0.19(5)	8(*f*)	1	0.898(4)	0.500(8)	0.493(5)	0.05[a]

[a] not refined
